# Acute exposure to a sublethal dose of imidacloprid and coumaphos enhances olfactory learning and memory in the honeybee *Apis mellifera*

**DOI:** 10.1007/s10158-012-0144-7

**Published:** 2012-11-17

**Authors:** Sally M. Williamson, Daniel D. Baker, Geraldine A. Wright

**Affiliations:** 1 Faculty of Medical Sciences, Centre for Behaviour and Evolution, Institute of Neuroscience, Newcastle University, Newcastle upon Tyne, NE1 7RU UK; 2 School of Biology, Newcastle University, Newcastle upon Tyne, NE1 7RU UK

**Keywords:** *Apis mellifera*, Olfactory learning, Imidacloprid, Coumaphos, Pesticide

## Abstract

The decline of honeybees and other pollinating insects is a current cause for concern. A major factor implicated in their decline is exposure to agricultural chemicals, in particular the neonicotinoid insecticides such as imidacloprid. Honeybees are also subjected to additional chemical exposure when beekeepers treat hives with acaricides to combat the mite *Varroa destructor*. Here, we assess the effects of acute sublethal doses of the neonicotinoid imidacloprid, and the organophosphate acaricide coumaphos, on honey bee learning and memory. Imidacloprid had little effect on performance in a six-trial olfactory conditioning assay, while coumaphos caused a modest impairment. We report a surprising lack of additive adverse effects when both compounds were administered simultaneously, which instead produced a modest improvement in learning and memory.

## Introduction

Honeybee populations are in decline in many countries. An important factor implicated in this decline is exposure to agricultural chemicals used to combat pests and fungi that bees experience when they pollinate flowering crops or plants near agricultural land (Dainat et al. 2011; Neumann and Carreck 2010; vanEngelsdorp et al. 2009). Systemic insecticides, such as the neonicotinoids, are of particular concern as they persist in pollen and nectar long after application (Rortais et al. 2005). Domesticated honeybees are also exposed to chemical acaricides administered by beekeepers within the colony to control infestations of the parasitic mite *Varroa destructor* (Rosenkranz et al. 2010). It has been suggested that combined exposure to both pesticides and acaricides may be more toxic to bees than exposure to a single toxic compound. Reasons for this include the same detoxification mechanisms being utilized in response to various different toxins: these mechanisms include cytochrome P450 enzymes and multi-drug resistance (MDR) xenobiotic transporters (Hawthorne and Dively 2011; Johnson et al. 2009). Another possibility is that combinations of pesticides and acaricides may have additive or synergistic effects on the nervous system, especially when they affect the same physiological targets (Gill et al. 2012; Laetz et al. 2009).

Successfully foraging for pollen and nectar requires that bees perform many sophisticated behaviours, including accurate navigation and associative learning and generalization. The neural circuits that govern olfactory and gustatory sensation and learning and memory are all mediated by cholinergic neurotransmission (Gauthier 2010). Neonicotinoid pesticides such as imidacloprid act as agonists of certain subtypes of insect nicotinic acetylcholine receptors (nAChRs), including those in the honeybee brain (Barbara et al. 2008; Buckingham et al. 1997). It is, therefore, perhaps unsurprising that imidacloprid has been shown to impair olfactory learning and memory in the honeybee (Decourtye et al. 2004a, b).

In addition to the neonicotinoids, other classes of pesticide which may directly disrupt cholinergic signalling include the carbamates and organophosphates, which act as inhibitors of acetylcholinesterase (Fukuto 1990). Acetylcholinesterase is present throughout the brain, and its disruption has been shown to directly affect olfactory learning and memory (Gauthier et al. 1992; Kreissl and Bicker 1989). In many parts of the world, neonicotinoids are replacing carbamates and organophosphates as the pesticide of choice for crop protection, due to their lower mammalian toxicity (Elbert et al. 2008). However, one particular organophosphate compound, coumaphos, is still of direct relevance to honeybee health: in the United States, coumaphos is used as an in-hive mite treatment and is known to accumulate in comb wax (Milani and Iob 1998; Mullin et al. 2010). Despite this, very little is known about the adverse effects of coumaphos on honeybees, or about the combined effects of coumaphos and neonicotinoids on complex honeybee behaviours coordinated by cholinergic signalling.

This study investigates the effects of an acutely administered dose of imidacloprid, coumaphos, and a mixture of the two compounds, on olfactory learning and memory in the honeybee. Bees rely on olfactory cues as a means of identifying flowers containing nectar (Wright et al. 2009). The aim of these experiments was to identify how a single, sublethal dose of these compounds and their combination affected performance during two tasks: a massed conditioning task where the bee must rapidly acquire information about the learned association on a short (30 s) inter-trial interval (ITI) schedule, and a spaced learning task with a 10 min inter-trial interval. It has been shown previously that the massed training task may be more difficult than the spaced, due to rapidly delivered stimuli disrupting the process of memory consolidation (Menzel et al. 2001). We examined performance during learning and recall to identify how exposure affected the processes involved in olfactory learning and memory.

## Methods

### Honeybees

Honeybee colonies (*Apis mellifera mellifera*) were obtained from stock of the National Bee Unit (York, UK) and maintained at Newcastle University. Foraging adult workers were collected in small plastic vials from a single colony during the period between June and August 2011. Approximately 80 bees were collected on each occasion to allow for incidental mortality; surviving bees were distributed equally between the different treatment groups. This was repeated until *n* > 30 bees for each treatment group and training type. The vials were placed on ice until the bees were immobile, then the bees were restrained in plastic harnesses and secured with tape. Restrained bees were fed 1M sucrose solution ad libitum by placing the bee’s mouthparts near a container of solution and allowing them to drink until they were full. The bees were left overnight without food to become sufficiently motivated to respond to training. This feeding regime was repeated 30 min after short-term memory (STM) testing at 10 min to keep the bees alive for the 24-h long-term memory (LTM) test.

### Pesticides

Imidacloprid and coumaphos were obtained in dry powder form at >99 % purity from Sigma-Aldrich. Imidacloprid was directly dissolved in 1M sucrose solution to a concentration of 1 μM. Coumaphos was dissolved in dimethyl sulfoxide (DMSO) to a concentration of 10 mM and then diluted with 1M sucrose to 1 μM. Preliminary experiments demonstrated that DMSO at concentrations of 0.1 % or less had no effect on olfactory learning (data not shown). Bees were fed 5 μl of either sucrose (control group) or pesticide solution 1 h prior to olfactory conditioning. Pesticide treatment groups were imidacloprid, coumaphos, and imidacloprid plus coumaphos: doses per bee were 1.28 ng imidacloprid, 1.81 ng coumaphos, and 1.28 ng imidacloprid plus 1.81 ng per bee coumaphos in the combined treatment group. These doses were chosen so that the acute dose administered in this study approximately matched the accumulated dosage delivered in a parallel subchronic study (Williamson and Wright, in review), where bees were fed either 10 or 100 nM solutions of the same compounds ad libitum for 4 days. The acute dosage used here matched the accumulated dosage of bees fed the 10 nM solution, which in turn was in the range of predicted imidacloprid consumption by forager bees over a similar time period (Rortais et al. 2005). An acute dose of 12.8 ng imidacloprid and/or 18.1 ng coumaphos (equivalent to accumulated consumption of 100 nM solutions) caused a paralysis of the bees which made them unable to extend their proboscis and participate in the experiment.

### Learning and memory experiments

An olfactory conditioning protocol based on the proboscis extension reflex (PER) was used to train the honeybees (Bitterman et al. 1983). The pairing of the conditioned stimulus (odour) and unconditioned stimulus (sucrose reward) was presented on a massed conditioning schedule (30 s) or a spaced conditioning schedule (10 min) as described by Menzel and colleagues (Menzel et al. 2001). The conditioned odour was 1-hexanol (Sigma-Aldrich), and the unconditioned stimulus was a reward of 0.4 μl of 1M sucrose solution. The odour stimulus was a 3 μl aliquot of the odorant applied to a strip of filter paper placed within a glass tube and attached to controlled air supply (Wright et al. 2007). The air supply was controlled to deliver an odour stimulus of 4-s duration. Each subject received 6 conditioning trials; each subject was tested with the conditioned stimulus and a novel odour (2-octanone, Sigma-Aldrich) for late short-term memory (10 min) and early long-term memory (24 h) (Menzel and Giurfa 1999). The order of presentation of the test odours was randomized across subjects.

### Statistical analysis

**Fig. 1:**
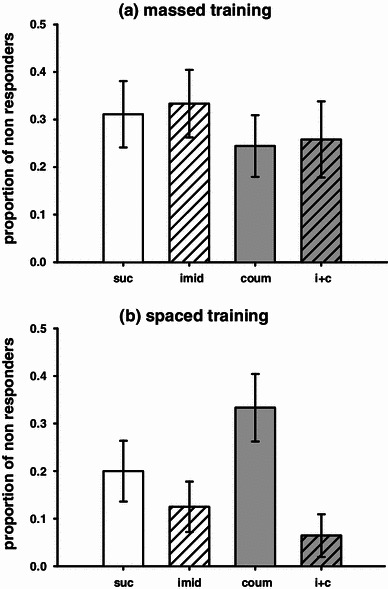
Proportion of bees which did not respond on any of the 6 conditioning trials with a learned response to the odour stimulus. Treatment groups are as follows: sucrose-fed control (*white bar*), imidacloprid (*white striped bar*), coumaphos (*grey bar*), and combined imidacloprid and coumaphos (*grey striped bar*). **a** Massed training (30 s ITI). None of the treatment groups were different to the controls, or to the other treatment groups. **b** Spaced training (10 min ITI). None of the treatment groups were different to the controls, but there are more non-responders in the coumaphos treatment group compared to both the imidacloprid and the imidacloprid plus coumaphos treatment groups (graphs show means ± SEMs, *n* ≥ 30 for all treatment groups)

The response of each subject to the odour stimulus during conditioning and testing was scored as a binary response (PER was scored as 1) and analysed using binary logistic regression (SPSS). The predictor variables were ‘treatment’ and ‘trial number’, and post hoc pairwise comparisons were used to perform comparisons between treatment groups. Logistic regression as a method of data analysis has been used in several previous studies of olfactory learning (Mustard et al. 2012; Wright et al. 2007). Subjects that responded to the conditioned stimulus on the first trial were excluded from the experiment. Subjects that exhibited PER in response to sucrose stimulation of the antennae but did not exhibit conditioned proboscis extension on any trial during conditioning to the conditioned stimulus were excluded from the acquisition and test data. This data were analysed separately using binary logistic regression, and the proportion of excluded non-responding bees for each treatment group can be seen in Fig. 1. For logistic regression analysis of the acquisition data, the first training trial (where response of included subjects was always 0) was excluded from the analysis to facilitate model fit. Mean values for the probability of response, and standard errors of the means, are reported for each treatment, dose, and odour presentation.

## Results

### Learning was impaired by coumaphos and enhanced by the combination of imidacloprid and coumaphos

Acute pesticide treatment affected the number of bees that could perform the spaced learning task, but not the massed learning task (Fig. 1, binary lreg, massed, χ_3_^2^ = 1.11, *P* = 0.774; spaced, χ_3_^2^ = 9.09*, P* = 0.028). For the bees that experienced spaced learning, the treatment group given coumaphos alone had significantly more non-responders than the imidacloprid, and imidacloprid plus coumaphos, treatment groups (imidacloprid, *P* = 0.017; imidacloprid plus coumaphos, *P* = 0.001), though none of the treatment groups were significantly different to the control.

**Fig. 2:**
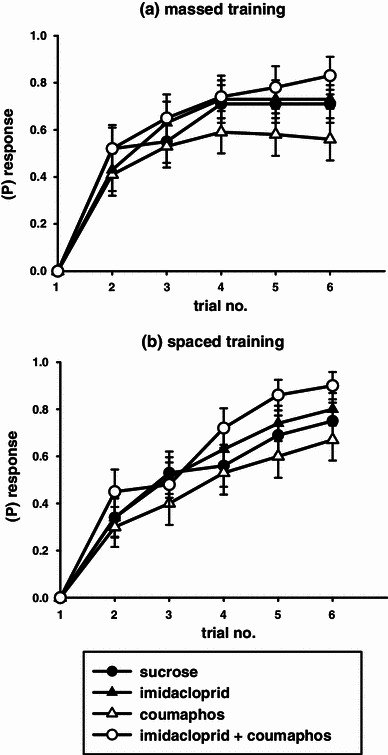
Acquisition curves for the six-trial training protocols (excluding the subjects from Fig. 1). Treatment group are as follows: sucrose-fed control (*black circle*), imidacloprid (*black triangle*), coumaphos (*white triangle*), and combined imidacloprid and coumaphos (*white circle*). **a** Massed training: coumaphos impairs olfactory learning. **b** Spaced training: combined treatment with both imidacloprid and coumaphos enhances olfactory learning (graphs show means ± SEMs, *n* ≥ 23 for all treatment groups)

Pesticide treatment also affected the bees’ ability to learn the conditioned stimulus–unconditioned stimulus (CS–US) association during both massed and spaced conditioning (Fig. 2, binary lreg, massed χ_3_^2^ = 9.77, *P* = 0.021; spaced, χ_3_^2^ = 10.38, *P* = 0.016). During massed training, coumaphos-treated bees showed impaired learning compared to the controls (*P* = 0.05), and also compared to the other treatment groups (imidacloprid, *P* = 0.027; imidacloprid plus coumaphos, *P* = 0.003). During spaced training, the combination of imidacloprid plus coumaphos actually enhanced learning when compared to the performance of the control group (*P* = 0.050) and the coumaphos treatment group (*P* = 0.001).

### The combination of imidacloprid and coumaphos enhance STM in massed conditioned bees

**Fig. 3:**
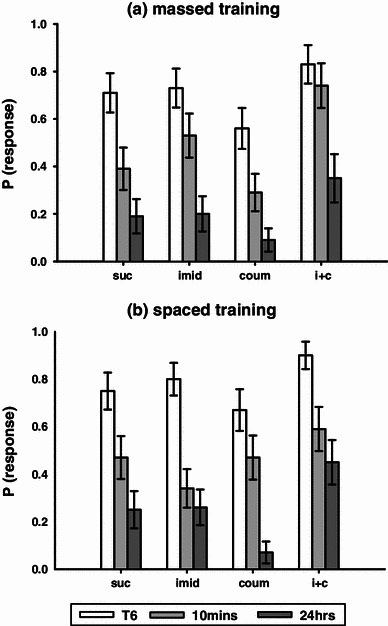
Memory test data: response rates to the CS are shown for the 6th training trial (*white bar*), the 10 min test of STM (*pale grey bar*), and the 24-h test of LTM (*dark grey bar*). **a** Massed training: the imidacloprid plus coumaphos treatment group showed enhanced STM relative to the control group and also performed better than the coumaphos treatment group for both STM and LTM. **b** Spaced training: coumaphos-treated bees showed impaired LTM relative to the controls and both other treatment groups (graphs show means ± SEMs, *n* ≥ 23 for all treatment groups)

The effects of pesticide treatment on olfactory STM were assessed in terms of response rate to the CS 10 min after olfactory conditioning. Acute treatment with pesticides affected STM in massed conditioned bees (Fig. 3a, binary lreg, χ_3_^2^ = 14.5, *P* = 0.002). This effect was an improvement in STM for bees that experienced acute treatment with both imidacloprid and coumaphos relative to the control group (*P* = 0.005) and relative to the coumaphos treatment group (*P* < 0.001). However, the STM of the subjects that experienced spaced conditioning was unaffected by acute pesticide application (Fig. 3b, binary lreg, χ_3_^2^ = 5.32*, P* = 0.150).

The effects of pesticide treatment on olfactory LTM were measured as the response rate to the CS 24 h after olfactory conditioning. Acute pesticide application influenced LTM for both the massed and the spaced conditioned bees (Fig. 3, massed, binary lreg, χ_3_^2^ = 15.1, *P* = 0.002; spaced, binary lreg, χ_3_^2^ = 11.0, *P* = 0.012). As observed during STM, bees acutely treated with the combination of imidacloprid and coumaphos and subjected to massed conditioning exhibited a significantly higher probability of responding during the 24-h recall test than the bees treated with coumaphos alone (*P* = 0.019). However, neither of these treatment groups responded significantly differently to the control group (imidacloprid plus coumaphos, *P* = 0.206; coumaphos, *P* = 0.221). For the spaced conditioning bees, however, the coumaphos treatment group showed impaired LTM relative to the control group (*P* = 0.040).

### Testing memory specificity: comparing responses to the CS and a novel odour

To confirm that the responses to the CS in the section above were an accurate assessment of memory formation, we also compared how the bees responded to both the CS and a novel odour presented during the tests for STM and LTM. The control subjects were always more likely to respond to the CS than the novel odour (massed, *P* = 0.005; spaced, *P* < 0.001).

**Fig. 4:**
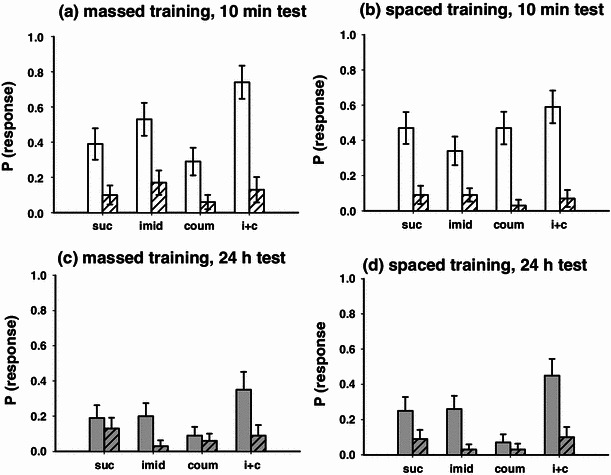
Assessing memory specificity using a novel odour test: response rates are shown for both conditioned (*solid colour bar*) and novel (*striped bar*) odours. Acute pesticide treatments did not affect STM specificity after either **a** massed or **b** spaced training, and all groups were able to discriminate between the two odours. Acute treatment with imidacloprid, or combined imidacloprid and coumaphos, enhanced LTM specificity after both **c** massed and **d** spaced training (*graphs* show means ± SEMs, *n* ≥ 23 for all treatment groups)

Acute pesticide exposure did not influence the bees’ ability to discriminate between the CS and a novel odour at the STM time point (Fig. 4a, b, massed, binary lreg, χ_3_^2^ = 3.98, *P* = 0.264); spaced, (binary lreg, χ_3_^2^ = 1.24, *P* = 0.744).

The specificity of olfactory LTM, however, was affected by acute pesticide treatment after both massed and spaced conditioning (Fig. 4c, d, massed, binary lreg, χ_3_^2^ = 7.84, *P* = 0.050; spaced, binary lreg, χ_3_^2^ = 7.55, *P* = 0.005). At the 24 h after conditioning, control bees had lost this specificity and did not respond to the CS significantly more than the novel odour (massed, *P* = 0.488; spaced, *P* = 0.090). However, treatment with either imidacloprid or combination of imidacloprid and coumaphos enhanced performance: these treatment groups responded to the CS significantly more often than to the novel odour (massed, imidacloprid *P* = 0.037, imidacloprid plus coumaphos *P* = 0.024; spaced, imidacloprid *P* = 0.004, imidacloprid plus coumaphos *P* = 0.001).

## Discussion

These experiments show that acute, sublethal coumaphos treatment impaired olfactory learning and memory in the honeybee, whereas acute administration of combined coumaphos and imidacloprid actually enhanced learning and memory. Imidacloprid did not have a strong effect on acquisition when administered alone. The specificity of the olfactory memory measured at 24 h after conditioning was also slightly improved by both imidacloprid and combined imidacloprid and coumaphos treatment, but unaffected by the other treatments.

Previous studies of imidacloprid’s influence on learning, however, have reported that higher acute doses (12 ng per bee) reduced the rate of responses of bees during olfactory learning (Decourtye et al. 2004a). In contrast, we found that an acute, sublethal dose of imidacloprid had no adverse effect on learning and memory at the dosage used here (1.28 ng/bee). However, pilot experiments performed in our own laboratory to determine dosage found that 12.8 ng per bee induced a paralysis, leaving the bees unable to extend their proboscis in response to either sucrose or odour stimulation; therefore, it may be difficult to discern whether learning, or proboscis extension, is actually impaired at high doses. That imidacloprid treatment slightly enhanced memory specificity and rescued the adverse effects of coumaphos treatment in our experiments is surprising. However, it has been demonstrated previously that activation of honeybee nAChRs using nicotine can enhance learning and memory while blocking nAChRs with antagonists impairs these processes (Gauthier et al. 2006; Lozano et al. 2001; Thany and Gauthier 2005). Imidacloprid and nicotine could cause learning and memory enhancement by amplifying excitatory input during olfactory stimulation when nAChRs in the mushroom bodies and antennal lobes are activated (Thany and Gauthier 2005). In the work presented here, imidacloprid particularly enhanced LTM, which may suggest it is specifically activating the α-bungarotoxin-sensitive receptors affecting long-term memory storage in mushroom body neurons (Gauthier et al. 2006; Jepson et al. 2006).

In the results we present here, acute coumaphos treatment caused a slight impairment of learning and memory processes. Other studies which have investigated the effects of acetylcholinesterase inhibitors on honeybee learning and memory have showed conflicting results: Shapira and colleagues found that metrifonate enhanced learning, which correlated with their observations that AChE levels were lower in bees which performed well in an olfactory learning test (Shapira et al. 2001). In contrast to this, and to our own data, Weick and colleagues found that acute coumaphos treatment had no effect on learning or memory, with just a small effect on odour discrimination (Weick and Thorn 2002). However, due to the difference in administration methods between this work and the work of Weick and Thorn (injection in hexane versus ingestion in sucrose syrup), the results are unlikely be directly comparable. Organophosphate compounds require metabolic activation to act as acetylcholinesterase inhibitiors (Fukuto 1990), and it is possible that injection, or even acute oral administration, may not allow enough time for the compound to be fully metabolized to the active form.

Imidacloprid and coumaphos both target cholinergic signalling, which led us to investigate whether an additive or synergistic effect would be observed when both compounds were administered together. It might have been predicted that both compounds would impair learning and memory more than either compound administered alone. However, a different and rather unexpected effect was observed, with imidacloprid treatment not only reversing the learning and memory deficits caused by coumaphos, but also enhancing memory relative to the control group. Imidacloprid is an agonist of only certain subpopulations of nAChRs in the brain, as it is well established that differing subunit combinations generate nAChRs with different pharmacological properties (Lansdell and Millar 2000). Coumaphos, however, has less specific effects, targeting all cholinergic signalling, via both nicotinic and muscarinic pathways (Chen 2012; Pohanka 2011). The importance of AChE activity in modulating learning and memory in the honeybee is well established, and previous studies have shown that low AChE activity, or acute AChE inhibitor treatment, correlate with enhanced learning performance (Gauthier et al. 1992; Guez et al. 2010; Shapira et al. 2001). Coumaphos alone may not have been sufficient to raise ACh levels enough to enhance cholinergic learning processes alone. One possible explanation is that inhibition of AChE would have elevated ACh and in combination with imidacloprid could have produced greater activation of the nAChRs in the antennal lobes and mushroom bodies, resulting an enhancement of learning and, therefore, better memory formation. This implies that this subset of receptors is important in the establishment and formation of long-term memory in the honeybee (Gauthier 2010).

The results presented here differ from our findings in a previous study, where a subchronic treatment regime with the same compounds was used. After prolonged exposure to sublethal doses of these same pesticides (fed ad libitum at a lower concentration, equating to approximately 1.28 or 12.8 ng per bee being consumed over 4 days), both imidacloprid and coumaphos impaired olfactory learning and memory (Williamson and Wright, in review). Differences between the effects of acute and chronic imidacloprid administration have previously been reported, with chronic administration increasing the toxicity, so the accumulated lethal dose over several days was much lower than the acute lethal dose (Suchail et al. 2001). These discrepancies highlight the need to interpret the effects of neurotoxic pesticides in the context of the experimental methods used, as long-term exposure to such substances has dramatically different influences on the way that the brain functions (Cresswell 2011). In the context of realistic field exposure, the dose of imidacloprid used here may exceed that found in nectar and pollen, but are within range of the levels of systemic insecticides found to accumulate in wax and stored food within a hive (Mullin et al. 2010; Rortais et al. 2005). The coumaphos exposure experienced by domesticated honeybees treated with this compound as an acaricide may be well in excess of the dosage used here, with levels in comb wax and stored pollen reported as reaching several thousand ppb (Mullin et al. 2010; Wu et al. 2011).

In summary, this study adds to the body of literature which addresses the effects of pesticides which affect cholinergic signalling on ecologically relevant aspects of honeybee behaviour. The marked differences between the results presented here, and our previous study using a longer treatment period, highlight the importance of assessing both acute and chronic effects of pesticide exposure. In the case of imidacloprid, a systemic insecticide, and coumaphos, an in-hive mite treatment, chronic, prolonged exposure is more representative of realistic exposures in the field (Halm et al. 2006; Wu et al. 2011). However, it is only by assessing the same biological parameters after both acute and chronic exposure regimes that the dramatic differences in sublethal effects can be highlighted. Although the mechanisms underlying these different effects are not yet fully understood, future studies where imidacloprid and coumaphos are applied directly to neuronal cultures or recombinant honeybee receptors will help to further our understanding of the exact mechanisms of pesticide action within the honeybee brain.
